# Remarks on Mr. Stevenson's Comment on Dr. Marshall Hall's View of the Vis Nervosa, and on a Case of Great Loss of Nervous Power Resulting from a Carious Tooth

**Published:** 1851-07

**Authors:** J. L. Levison

**Affiliations:** Brighton.


### ARTICLE XVIII.

*Remarks on Mr. Stevenson's comment on Dr. Marshall Hall's, view of the Vis Nervosa, and on a case of great loss of nervous power resulting from a carious tooth.* By J. L. Levison, Esq., Brighton.

In a note by Mr. Stevenson, in the Lancet of June 22, on the statement of Dr. Marshall Hall, "that the vis nervosa, or power of the spinal nervous system, is not electricity in any of its known forms or modifications," he asserts that the doctor's views are altogether gratuitous, and cites Mr. Crosse's experiments as conclusive evidence of a contrary theory, but which, to my mind, to use a legal phrase, was not "proven," because similarity of phenomena does not necessarily lead to the inference, that there must be an actual identity. For, with all deference to Mr. Stevenson, he has not demonstrated by evidence that the vis nervosa of insect life is one and the same with that which depends on a cerebro-spinal system of the higher animals, including man. I therefore venture an hypothesis, that there exists some modification or difference in the electricity (vis nervosa) of different animals? My reasons are founded on an accidental discovery I made a few years since in the Adelaide Gallery. It was whilst taking shocks from a series of powerful magnets, (the electro-magnetism,) and which affected me in a very different manner from the shocks taken from either a cylindrical or a plate electrical machine. From the latter there is experienced, immediately on completing the electric circle by the hand, a transmission of the fluid through the whole range of the spinal nervous system; whilst the shocks from the magnets were only felt from the fingers to the elbow-joints. So that after repeatedly verifying the accuracy of these observations, the conclusions appeared to warrant the supposition, that however similar the phenomena of electricity and electro-magnetism, they were not exactly identical; and having reflected on the many anomalies of the nervous system, showing such marked difference in health and disease, I am disposed to think with Dr. M. Hall, "that the power (vis nervosa) of the spinal nervous system is not electricity in any of its known forms or modifications," but that this vis nervosa is something *sui generis*, and therefore cannot be regarded as identical with either electricity or galvanism, or electro-magnetism, or with electro-thermo-magnetism.

The reflex action of the nervous system, so ably elucidated by Dr. Marshall Hall, seems to favor such an inference, and to warrant the supposition, that whatever may be the peculiar nature or properties of the vis nervosa, it is subject to modification, in different diseases, according to the proximate cause of the primary disturbing influences—by the kind of tissues or organs involved, and by the peculiar idiosyncrasy of individuals. For how, otherwise, can we account for so many sympathetic disturbances, as, for instance, in ordinary caries of a tooth. The neuralgic pains may affect the ears in one person —the eyes of another, and, in the third, there may be violent throbbing of the arteries, with occasional intense suffering along the whole course of the maxillary branch of the fifth pair of nerves.

As an example of some remarkable disturbance from the presence of a carious tooth, and its irritation of surrounding textures, I may cite the following case:

Miss ——, a young lady, was brought in a carriage to my residence, to have her mouth examined. On being removed, she was supported by a lady on one side, and a man-servant on the other, and her entire muscular system seemed paralyzed. Her legs trailed on the ground, like useless appendages; her arms, when raised, fell powerless immediately, when unsupported; and even the muscles of the tongue were paralyzed, and in her efforts to speak, this important organ remained in a quiescent state. On examining the mouth, I perceived a *dens sapientia* of the lower jaw very carious, and deeply imbedded in the temporal muscle, just below the ridge of the coronoid process, in which locality there was extensive inflammation. I suggested the removal of the latter tooth; and, though I had anticipated some advantage from the operation, the actual results astonished me. She instantly obtained the free motion of her tongue, which she immediately used to communicate an important fact—viz. "that ever since the time the tooth I had extracted had been making its way through the gum, she could date the gradual loss of power over her limbs," &c. I saw her about a month afterwards; she could then use her hand and arm: she was writing a letter! Since then, I have not seen what further progress she has made.

In this case, we had palpable proof that the phenomena could only be explained by assuming that the local irritation (shown by the great vascularity of the part) had, in the first instance, affected the maxillary branch of the fifth nerve, implicating the trunk of the nerve itself, and ultimately communicating this disturbed condition, by reflex action, to the spinal system. From this and similar cases, I think that there must be some modification in the vis nervosa, depending on some predisposition, local or general, or from some peculiar constitutional condition; for if such were not the case, why does not every tooth similarly affected produce, in all cases, uniformly similar consequences, in obedience to the law—"Like causes produce like effects?"—*Lancet.*

